# Characterization of the complete mitochondrial genome of *Rhododendron simsii* var. *putuoense* and its comparative evolutionary analysis

**DOI:** 10.3389/fpls.2026.1955008

**Published:** 2026-09-14

**Authors:** Hong Zhu, Chunlei Yue

**Affiliations:** 1 Zhejiang Academy of Forestry, Research Centre for Zhejiang Wetland, Hangzhou, China; 2 Key Laboratory of National Forestry and Grassland Administration on Ecosystem Conservation and Restoration in the Yangtze River Delta, Hangzhou, China

**Keywords:** comparative genomics, interorganellar gene transfer, mitochondrial genome annotation, phylogenetic evolution, *Rhododendron simsii* var. *putuoense*

## Abstract

*Rhododendron simsii* var. *putuoense* is a coastal-endemic plant with significant ecological and genetic resource value. Although its chloroplast genome (cpDNA) was recently characterized, its mitochondrial genome (mtDNA) remains unexplored. Here, we report the first complete assembly and annotation of the *R. simsii* var. *putuoense* mtDNA using PacBio HiFi long-read sequencing. The genome sequence is 658,161 bp in length, organized as two subgenomic linear molecules (a major one of 633,105 bp and a minor one of 25,056 bp), and encodes 33 protein-coding genes (PCGs), 26 tRNAs, three rRNAs and 323 ORFs. Repeat analysis revealed a predominance of dispersed repeats (72%), alongside 128 SSRs and 20 tandem repeats. The mtDNA exhibits a strong A/U-ending codon usage bias, with 463 RNA editing sites predicted. Six homologous segments (0.91% of the mtDNA) provide evidence of interorganellar gene transfer (IGT). Notably, pairwise *K*_a_/*K*_s_ analysis revealed an elevated ratio in the *matR* gene, suggesting at lineage-specific evolutionary dynamics that warrant further site-specific codon model testing. Phylogenetic and synteny analyses further confirmed a close relationship with *R. simsii*, while revealing structural divergence consistent with ecological specialization. Overall, these findings deepen our understanding of mitochondrial genomic diversity within *Rhododendron* and establish a genetic framework for conserving lineage-specific resources in this ecologically significant variety.

## 1 Introduction

The study of organelle genomes is of great significance for understanding plant genetics, evolution, and adaptation (Gualberto and Newton, 2017; Møller et al., 2021). As the central organelle for energy metabolism, the mitochondrion harbors key genes related to the respiratory chain (Nolfi-donegan et al., 2020). Compared with the high-density coding of nuclear genomes and the relative conservation of chloroplast genomes (cpDNA), the most prominent features of plant mitochondrial genomes (mtDNA) are their highly dynamic structures and diverse origins of content (Palmer et al., 2000). With the advancement of high-throughput sequencing technologies and the deepening of comparative genomics, analyzing the mtDNA of non-model plants has become a critical entry point for exploring plant adaptive evolution (Zeng et al., 2024; Zhu and Lv, 2025; Zhang et al., 2025).

As one of the most evolutionarily active groups within the Ericaceae family (Shrestha et al., 2018), the genus *Rhododendron* has long been a challenge in plant systematics due to its complex taxonomy system (Mo et al., 2022; Zhu et al., 2025a). Currently, organelle genome research in this genus focuses predominantly on cp genomes (Ma et al., 2024; Zhou et al., 2024; Zhu et al., 2025b), which provide essential evidence for phylogenetic reconstruction. However, the structural characteristics, evolutionary pressures, and their relationship with ecological adaptation of the mtDNA within this genus have not yet been systematically resolved. This gap, to some extent, restricts a comprehensive understanding of the evolutionary mechanisms of *Rhododendron.*

*Rhododendron simsii* var. *putuoense*, a variety of *R. simsii*, is endemic to the coastal islands of East China, with a narrow geographical range (Li et al., 2010). Its unique coastal habitat, characterized by environmental stressors such as high salinity and strong winds, has shaped distinct evolutionary traits at the genomic level (Zhu et al., 2026). Although its cp genome was recently characterized (Zhu et al., 2025b), its mtDNA remains unexplored, and a comprehensive analysis of its organellar genomes remains lacking.

In this study, we performed the first assembly and annotation of the *R. simsii* var. *putuoense* mtDNA, leveraging the high-quality, chromosome-level nuclear genome (Zhu et al., 2026) and cp genomes previously sequenced for this taxon. By employing comparative genomics, collinearity analysis, phylogenetic reconstruction, and selective pressure analysis, we systematically revealed its genomic structure, evolutionary pressures, and phylogenetic relationships with closely related species. These findings aim to provide important baseline evidence for understanding both the structural plasticity and evolutionary dynamics of *Rhododendron* mtDNA, while offering preliminary genomic insights into potential adaptive divergence. Overall, this work establishes a robust genetic foundation for the conservation and utilization of this taxon.

## 2 Materials and methods

### 2.1 Raw data collection and filtering

The raw data used for the assembly of the *R. simsii* var. *putuoense* mt genome were extracted from the whole-genome sequencing (WGS) dataset of this taxon (Zhu et al., 2026), which is publicly available in the National Center for Biotechnology Information (NCBI) database under the accession number JBQRKF000000000 (BioProject ID: PRJNA1305678). According to the previously published study by our research group, the samples used in this study were collected from wild mature individuals at Wulei Mountain, Dinghai District, Zhoushan City, Zhejiang province (coordinates: 29.43°N, 122.20°E; altitude: 359 m) in March 2025. The sample was identified and collected by the authors, and a specimen was deposited in the Herbarium of Zhejiang Academy of Forestry (HZJAF, contact: Hong Zhu, Email: 1107401987@qq.com) under the voucher number HZ2025-DH-05.

### 2.2 Mt genome assembly and annotation

The PacBio HiFi sequencing raw data were assembled using PMAT software (v2.0.2, with the parameter -g 5G) (Bi et al., 2024). The resulting assembly was visualized and manually adjusted using Bandage (v0.8.1) (Wick et al., 2015) to generate a preliminary draft sequence. Subsequently, the HiFi data were aligned to the assembled sequence using Minimap2 software (Li, 2018), and the assembly was polished using NextPolish (Hu et al., 2020) to obtain the final sequence, followed by corresponding adjustments of the graphical fragment assembly (GFA) file. The annotation of the mt genome structure was conducted in several sequential steps. Protein-coding genes (PCGs), tRNAs, and rRNAs were annotated using the PMGA online platform (http://47.96.249.172:16084/annotate.html) and further refined through manual correction to obtain the final annotation results. Open reading frames (ORFs) with a minimum length threshold of 300 bp were identified using the NCBI ORF Finder (http://www.ncbi.nlm.nih.gov/gorf/gorf.html). After excluding redundant sequences and those overlapping with known genes, the ORFs were annotated by alignment against the NCBI non-redundant (nr) protein database. Finally, the physical map of the mt genome was generated using OGDRAW (v1.3.1) (https://chlorobox.mpimp-golm.mpg.de/OGDraw.html).

### 2.3 RNA editing site analysis and codon usage of PCGs

RNA editing sites were predicted based on the sequences of all protein-coding genes (PCGs) encoded by the mt genome. Specifically, cytosine (C)-to-uracil (U) RNA editing sites within the mt PCGs were identified using the Deepred-Mt tool (Edera et al., 2021). To ensure accuracy, only sites with a probability value greater than 0.9 were retained for subsequent analysis. The protein-coding sequences of the mtDNA were extracted using PhyloSuite (v1.1.16) (Zhang et al., 2020). Codon usage bias and relative synonymous codon usage (RSCU) were calculated using MEGA (v7.0) (Kumar et al., 2016). An RSCU value > 1 indicates that a codon is preferred, while an RSCU value < 1 indicates lower usage frequency, and RSCU = 1 represents no usage bias.

### 2.4 Analysis of repetitive sequences and interorganellar gene transfer

Repetitive sequences, including simple sequence repeats (SSRs), tandem repeats, and dispersed repeats, were identified using various bioinformatics tools. Specifically, SSRs were identified using MISA (v1.0), tandem repeats were detected via Tandem Repeats Finder (v4.09) (http://tandem.bu.edu/trf/trf.submit.options.html), and dispersed repeats were characterized using BLASTN (v2.10.1) (Altschul et al., 1990). All identified repeats were visualized using Circos (v0.69-5).

The cp genome of *R. simsii* var. *putuoense* (GenBank accession number: PV614722) had been previously published by our research group and was retrieved from the NCBI database. Homologous sequences between the cp and mt genomes were identified using BLASTN (v2.10.1) with a similarity threshold of 70% and an E-value of 1e-5. The results of the inter-organellar gene transfer (IGT) analysis were visualized using Circos (v0.69-5).

### 2.5 *K*_a_/*K*_s_ and nucleotide diversity (pi) analysis

Homologous gene pairs were extracted by grouping species in a pairwise manner. These gene pairs were subsequently aligned using MAFFT (v7.50) (Katoh and Standley, 2013). Following alignment, the *K*_a_ and *K*_s_ values for each gene pair were calculated using KaKs_Calculator (v2.0) (Wang et al., 2010), employing the MLWL method. The *K*_a_/*K*_s_ ratios for each gene pair were then statistically analyzed and visualized via box plots. For nucleotide diversity analysis, global alignments of homologous gene sequences from different species were performed, and the nucleotide diversity (pi) for each gene was calculated using DnaSP (v5) (Librado and Rozas, 2009).

### 2.6 Phylogenetic and synteny analysis

To investigate the evolutionary position of *R. simsii* var. *putuoense*, a comprehensive analysis of phylogeny and synteny was performed on this taxon and its representative related species. Complete mt genome sequences of 22 species from seven angiosperm families (Ericaceae, Actinidiaceae, Theaceae, Ebenaceae, Primulaceae, Symplocaceae and Aquifoliaceae) were retrieved from the NCBI database. Two *Ilex* species from the Aquifoliaceae family, *I. rotunda* (OR666441.1) and *I. macrocarpa* (OR066215.1), were used as outgroups. A Maximum Likelihood (ML) phylogenetic tree was reconstructed based on the coding sequences (CDS). Multiple sequence alignment was performed using MAFFT (v7.50) (Katoh and Standley, 2013), and the aligned sequences were concatenated. Subsequently, the sequences were trimmed using trimAl (v1.4.rev15) (Capella-gutiérrez et al., 2009) with the parameter “-gt 0.7”. The substitution model was predicted using jModelTest 2.1.10 (https://github.com/ddarriba/jmodeltest2), which identified the GTR model as the best-fit model. The ML tree was then constructed using RAxML v8.2.10 (https://cme.h-its.org/exelixis/software.html) under the GTRGAMMA model with 1,000 bootstrap replicates. To visualize the genomic relationships among these species, genome-to-genome alignments were performed using MUMmer (v4.0.0beta2) (Kurtz et al., 2004) with the “--maxmatch” parameter, and dot plots were generated.

## 3 Results

### 3.1 Basic characteristics of the *R. simsii* var. *putuoense* mtDNA

*R. simsii* var. *putuoense* was sequenced using the HiFi sequencing mode of the PacBio Sequel II platform. After processing the raw data, a total of 1,622,590 high-quality HiFi reads were obtained, yielding 34.27 Gb of data. The average read length was 21,119 bp, and the N50 was 21,174 bp. The assembled mtDNA of *R. simsii* var. *putuoense* has a total length of 658,161 bp and exhibits a multi-branched conformational structure with a GC content of 45.84% (**Figure 1a**). It consisted of two linear subgenomic molecules (a major and a minor molecule), with lengths of 633,105 bp (GC content: 45.83%) and 25,056 bp (GC content: 46.07%), respectively (**Figures 1b, c**).

**Figure 1:**
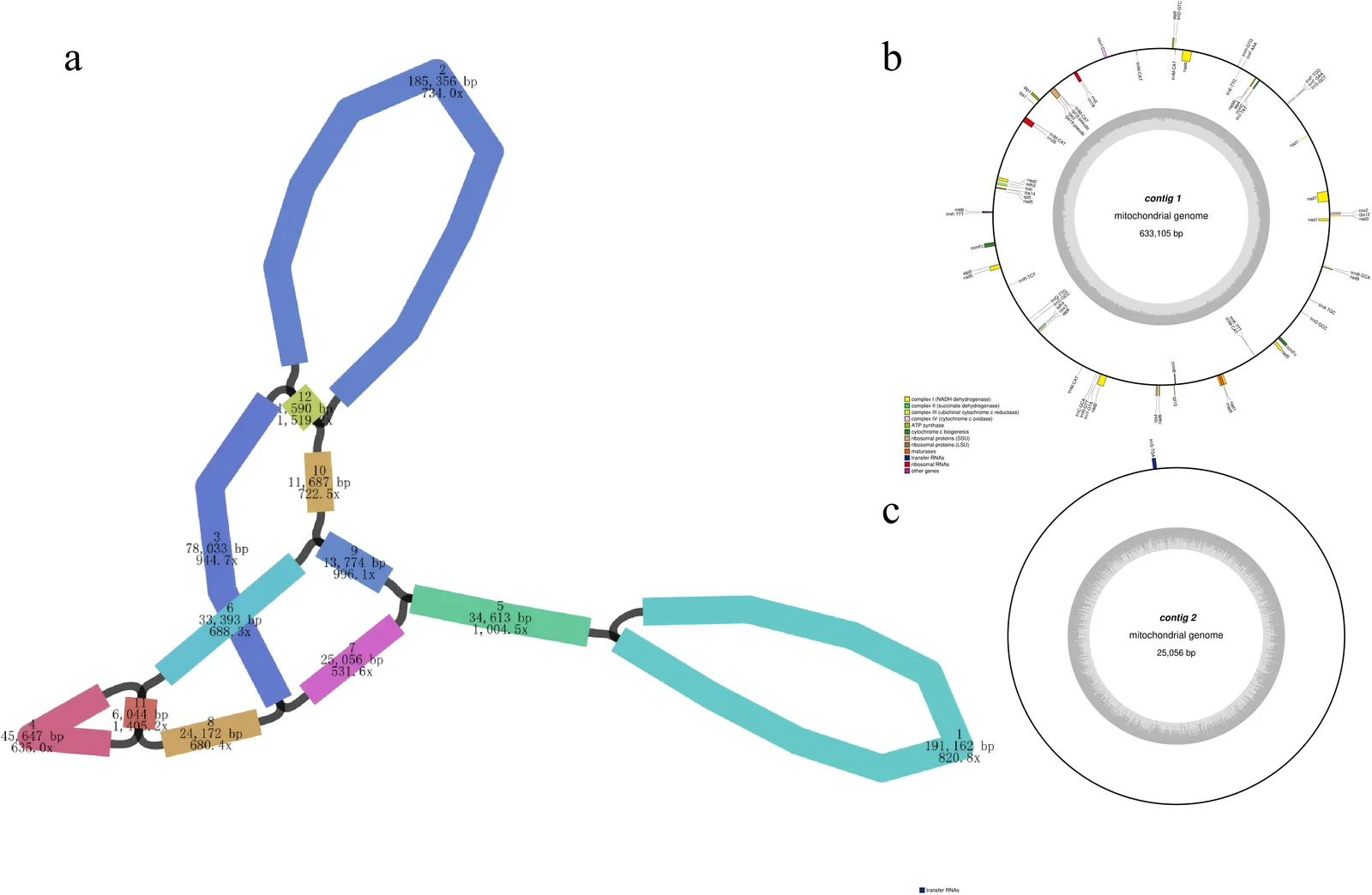
The assembly result of the *R. simsii* var. *putuoense* mtDNA. **(a)** The assembly graph displaying a multi-branched conformational structure; **(b, c)** Circular maps of the two independent linear subgenomic molecules, designated as contig 1 (major molecule, 633,105 bp) and contig 2 (minor molecule, 25,056 bp), respectively.

A total of 64 genes were annotated in the linear mtDNA of *R. simsii* var. *putuoense*, including 26 tRNA genes, three rRNA genes, two pseudogenes, and 33 PCGs. Additionally, 323 ORFs were identified. The 33 PCGs can be classified into 11 categories based on their functions: seven categories belong to core genes related to the core respiratory chain, including ATP synthase (5 genes), cytochrome c biogenesis (4 genes), ubiquinol-cytochrome c reductase (1 gene), cytochrome c oxidase (3 genes), maturases (1 gene), transport membrane protein (1 gene), and NADH dehydrogenase (9 genes). The remaining four categories are non-core functional genes, including large ribosomal proteins (LSU) (3 genes), small ribosomal proteins (SSU) (6 genes), succinate dehydrogenase (2 genes), and ribosomal RNAs (3 genes) (**Table 1**).

**Table 1:** Mt gene annotation of *R. simsii* var. *putuoense.*.

Group of genes	Names of genes
ATP synthase	*atp1*, *atp4*, *atp6*, *atp8*, *atp9*
Cytochrome c biogenesis	*ccmB*, *ccmC*, ccmFc^*^, *ccmFn*
Ubiquinol cytochrome c reductase	*cob*
Cytochrome c oxidase	*cox1*, *cox2*, *cox3*
Maturases	*matR*
Transport membrane protein	*mttB*
NADH dehydrogenase	nad1^****^, nad2^****^, *nad3*, nad4^**^, *nad4L*, nad5^****^, *nad6*, *nad*7^****^, *nad9*
Large ribosomal proteins (LSU)	^#^*rpl16*, *rpl10*, *rpl5*
Small ribosomal proteins (SSU)	^#^*rps19*, *rps1*, *rps12*, rps14^*^, rps3^*^, *rps4*
Succinate dehydrogenase	*sdh3*, *sdh4*
Ribosomal RNAs	*rrn18*, *rrn26*, *rrn5*
Transfer RNAs	*trnA*-TGC^*^, *trnC*-*GCA*, *trnD*-*GTC*, *trnE*-*TTC*, *trnF*-AAA^*^, *trnF*-*GAA*, *trnG*-*GCC*^(2)^, *trnH*-*GTG*, *trnI*-*TAT*, *trnK*-*TTT*, *trnK*-TTT^*^, *trnM*-*CAT*^(6)^, *trnN*-*GTT*, *trnP*-*TGG*, *trnQ*-*TTG*, *trnR*-TCT^*^, *trnS*-*GCT*, *trnS*-*TGA*, *trnW*-*CCA*, *trnY*-*GTA*

*indicates intron number; #indicates pseudogene; (2) indicates the number of copies of multi-copy genes.

### 3.2 Analysis of RNA editing sites

In total, 463 RNA editing sites were predicted across the 33 mt PCGs. Among them, the *mttB* gene possessed the highest number of RNA editing sites (39), followed by *ccmB* and *nad2*, each containing 38 RNA editing sites (**Figure 2**). Among the predicted editing events, 11.76% resulted in silent hydrophilic substitution (hydrophilic-to-hydrophilic), while 30.72% retained hydrophobicity (hydrophobic-to-hydrophobic). However, 49.67% of the amino acids transitioned from hydrophilic to hydrophobic, while 7.63% shifted from hydrophobic to hydrophilic. Additionally, 0.22% of the sequences resulted in premature termination of the coding process (stop codons).

**Figure 2:**
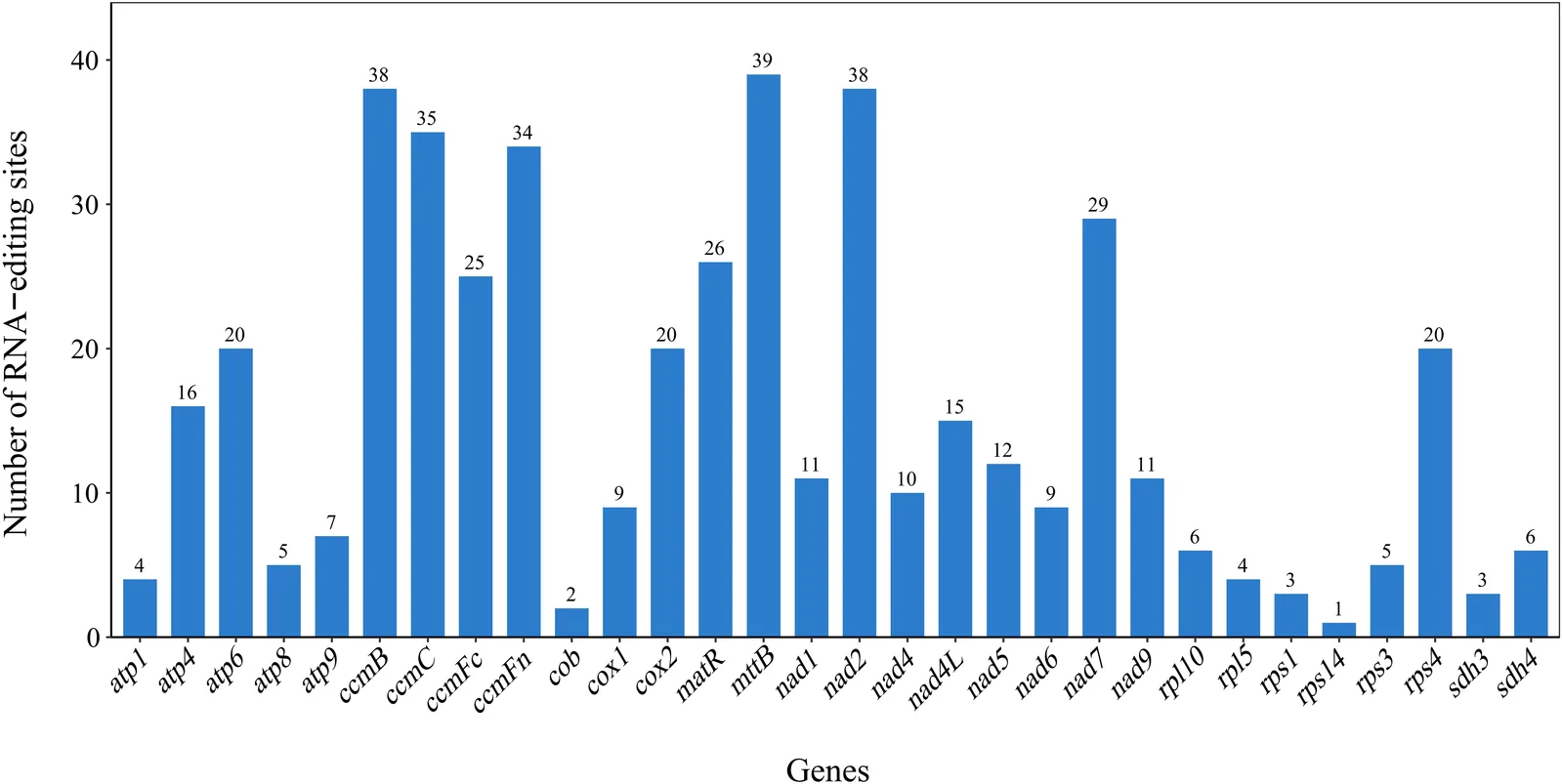
Distribution of RNA editing sites among *R. simsii* var. *putuoense* mt protein-coding genes.

### 3.3 Analysis of codon usage bias

A total of 9,987 codons were detected across all genes. Leucine (Leu), encoded by six synonymous codons, was the most abundant amino acid in this mtDNA, with 1,142 codons (11.43%). It was followed by serine (Ser) with 900 codons (9.01%) and arginine (Arg) with 617 codons (6.18%). In contrast, tryptophan (Trp) accounted for only 1.54% of the total amino acids. The RSCU values ranged from a maximum of 1.73 (UUA) to a minimum of 0.364 (UAG). Thirty-one codons had RSCU values greater than 1, indicating that these codons are used more frequently than other synonymous codons. Among these, 28 codons (90.32%) ended with A or U, while only three ended with C or G. This demonstrates a clear preference for A/U-terminated codons in the frequently used codons of the *R. simsii* var. *putuoense* mtDNA (**Figure 3**).

**Figure 3:**
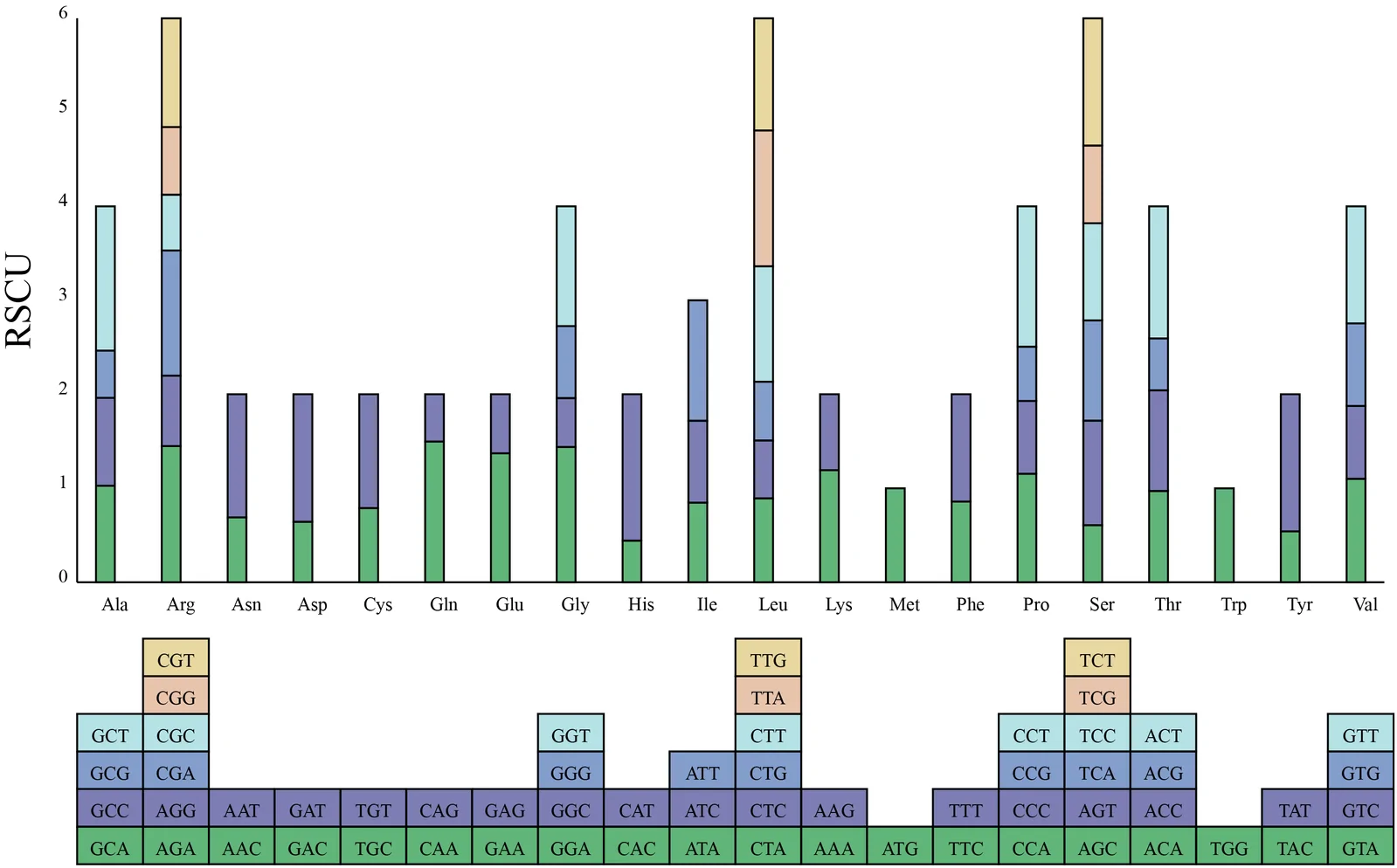
Relative synonymous codon usage (RSCU) histogram of *R. simsii* var. *putuoense* mt genome.

### 3.4 Analysis of repeat sequences

A total of 527 repetitive sequences were identified in the *R. simsii* var. *putuoense* mtDNA. Dispersed repeats were the most predominant, accounting for 379 (72%) of the total, including 170 pairs of forward repeats (F) and 209 pairs of palindromic repeats (P). Simple sequence repeats (SSRs) were the next most common, with 128 (24%) identified. Tandem repeats were the least frequent, representing only 20 (4%) of the total repetitions (**Figure 4**).

**Figure 4:**
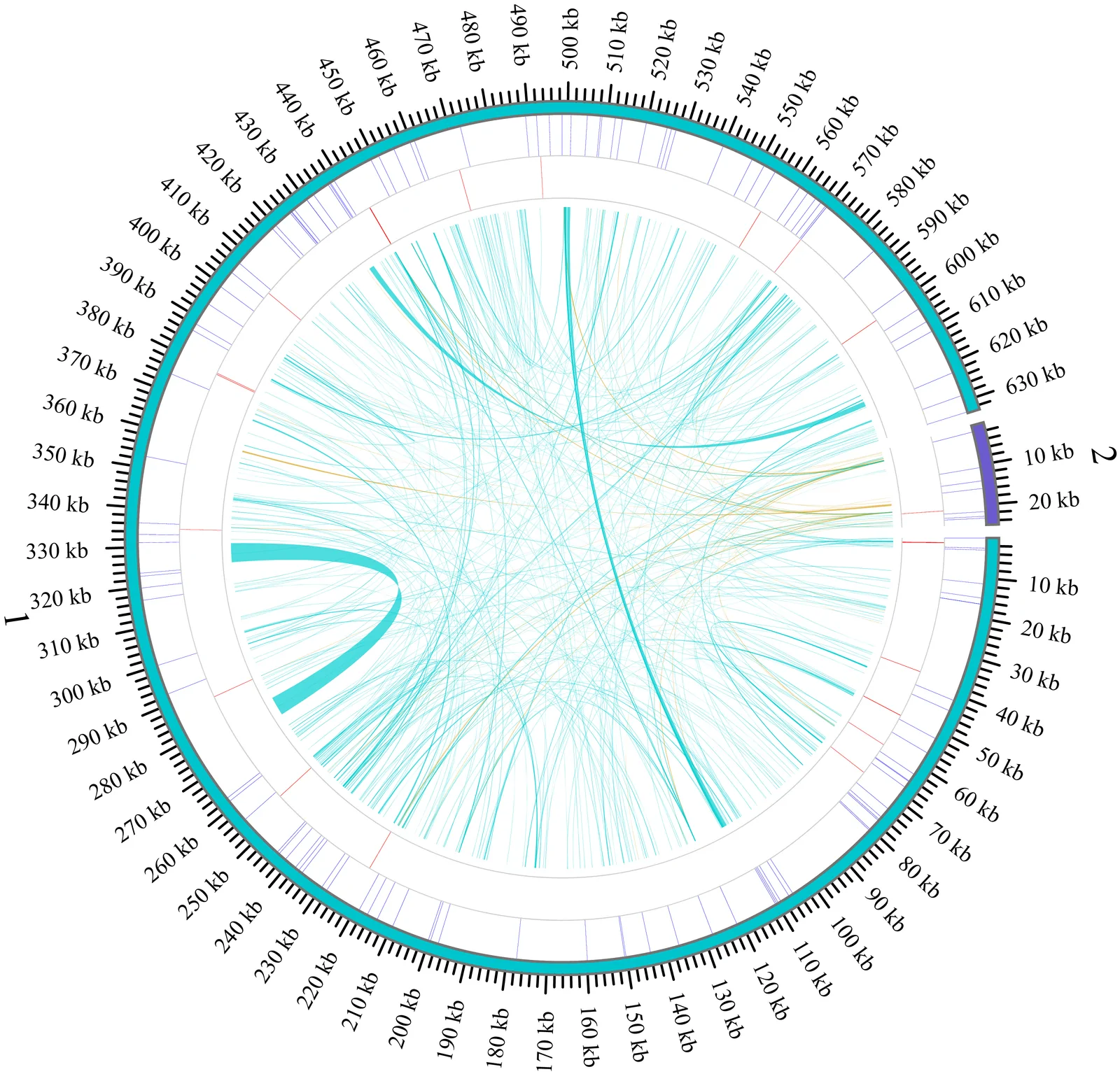
The distribution of repeats in *R. simsii* var. *putuoense* mtDNA. The outermost circle represents the mt genome sequence, and from outside to inside are simple sequence repeats, tandem repeats and dispersed repeats, respectively.

### 3.5 Analysis of homologous segments between the mt and cp genomes

A total of six homologous segments were identified between the mt and cp genomes, with a total length of 5,967 bp, accounting for 0.91% of the mtDNA. Among these, five segments were entirely located within the homologous sequences and were all tRNA genes (*trnA*-*TGC*, *trnW*-*CCA*, *trnD*-*GTC*, *trnN*-*GTT* and *trnM*-*CAT*). One segment was partially located (44.51%) within the homologous sequence, which belongs to the ribosomal RNA gene *rrn18* (**Figure 5**).

**Figure 5:**
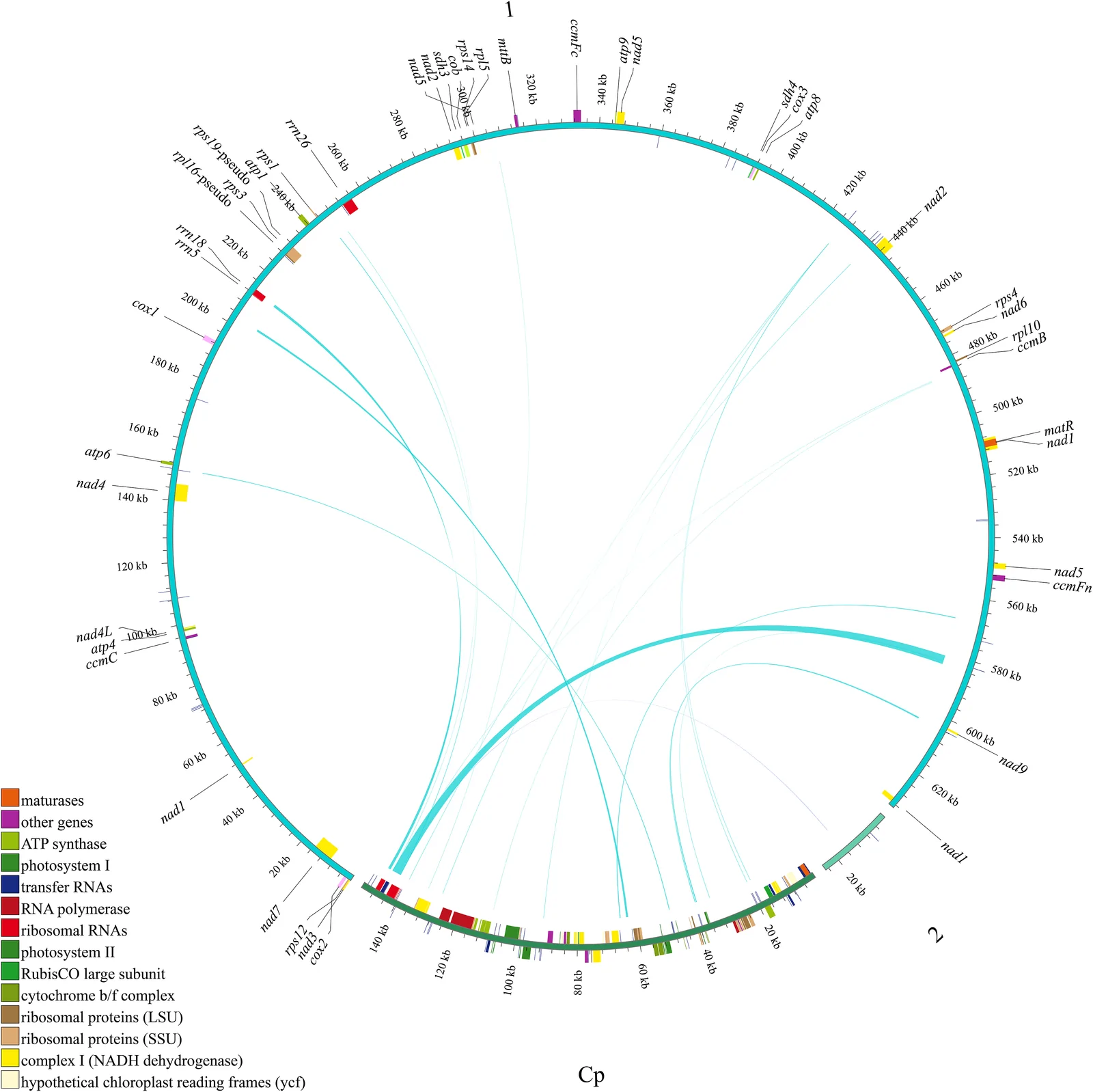
Interorganellar sequence transfer between cp and mtDNA of *R. simsii* var. *putuoense*.

### 3.6 *K*_a_/*K*_s_ analysis

In this study, the *K*_a_/*K*_s_ ratios were calculated for the shared PCGs among five *Rhododendron* species: *R. simsii* (MW030508.1), *R.* × *pulchrum* (OM283814.1), *R. decorum* (OQ450179.1), *R. vialii* (PQ537097.1) and *R. nivale* subsp. *boreale* (PQ537104.1). When comparing the mtDNA of *R. simsii* var. *putuoense* with *R. simsii*, the overall *K*_a_/*K*_s_ value was 0.65, with three PCGs exhibiting *K*_a_/*K*_s_ < 1. Compared to *R.* × *pulchrum*, the *K*_a_/*K*_s_ value was 0.61, with three PCGs showing *K*_a_/*K*_s_ < 1. Relative to *R. decorum*, the *K*_a_/*K*_s_ value was 0.47, and 13 PCGs had *K*_a_/*K*_s_ < 1. In comparison with *R. vialii*, the *K*_a_/*K*_s_ value was 0.32, with nine PCGs showing *K*_a_/*K*_s_ < 1, and one PCG showing *K*_a_/*K*_s_ > 1. Compared to *R. nivale* subsp. *boreale*, the *K*_a_/*K*_s_ value was 0.31, with 13 PCGs showing *K*_a_/*K*_s_ < 1. Notably, almost all *K*_a_/*K*_s_ ratios were less than 1, indicating that most PCGs have undergone purifying selection during evolution. In contrast, one gene (*matR*) exhibited a *K*_a_/*K* ratio > 1, pointing to a potential signature of elevated evolutionary rate or positive selection (**Figure 6**).

**Figure 6:**
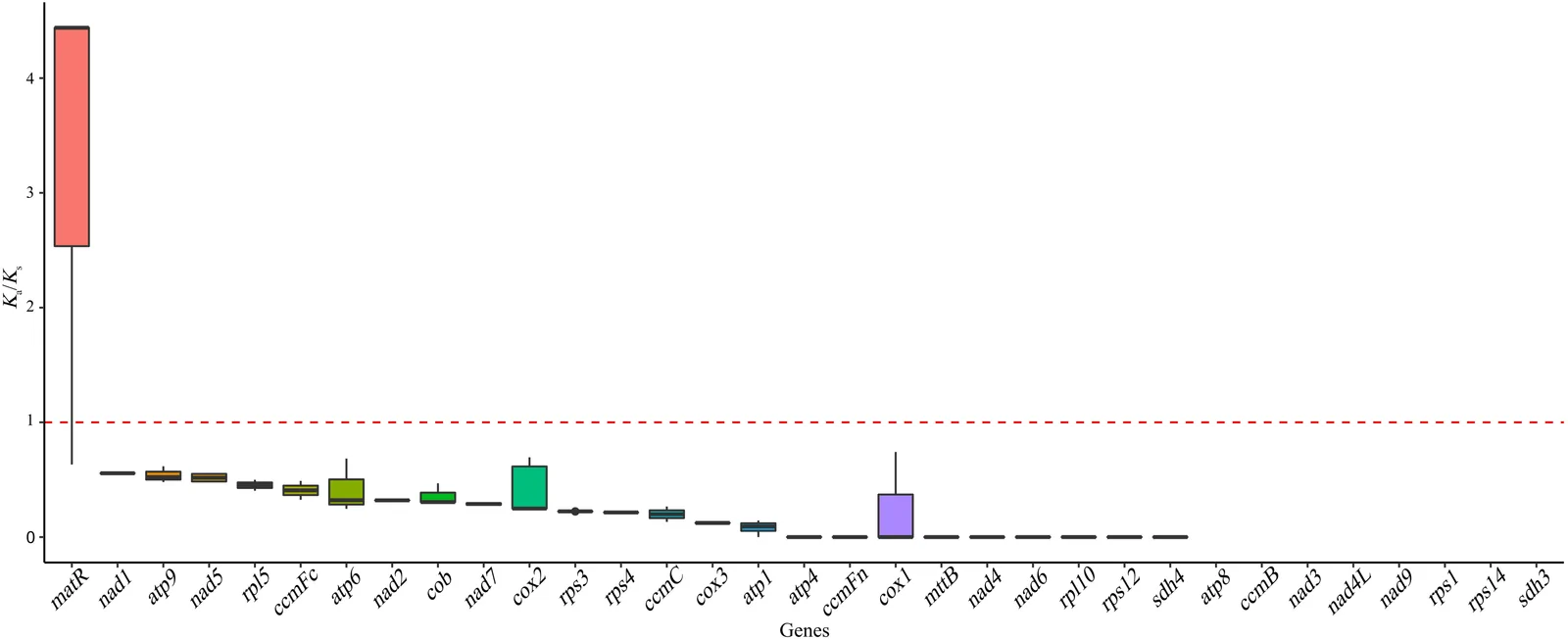
Boxplots of the pairwise *K*_a_/*K*_s_ values among all shared mt genes of the 6 *Rhododendron* species.

### 3.7 Phylogenetic and synteny analysis

The phylogenetic analysis of mt genomes showed that *R. simsii* var. *putuoense* clustered with *R. simsii* and *R.* x *pulchrum* in an independent subclade (bootstrap value = 100%), indicating an extremely close phylogenetic relationship among the three taxa (**Figure 7**).

**Figure 7:**
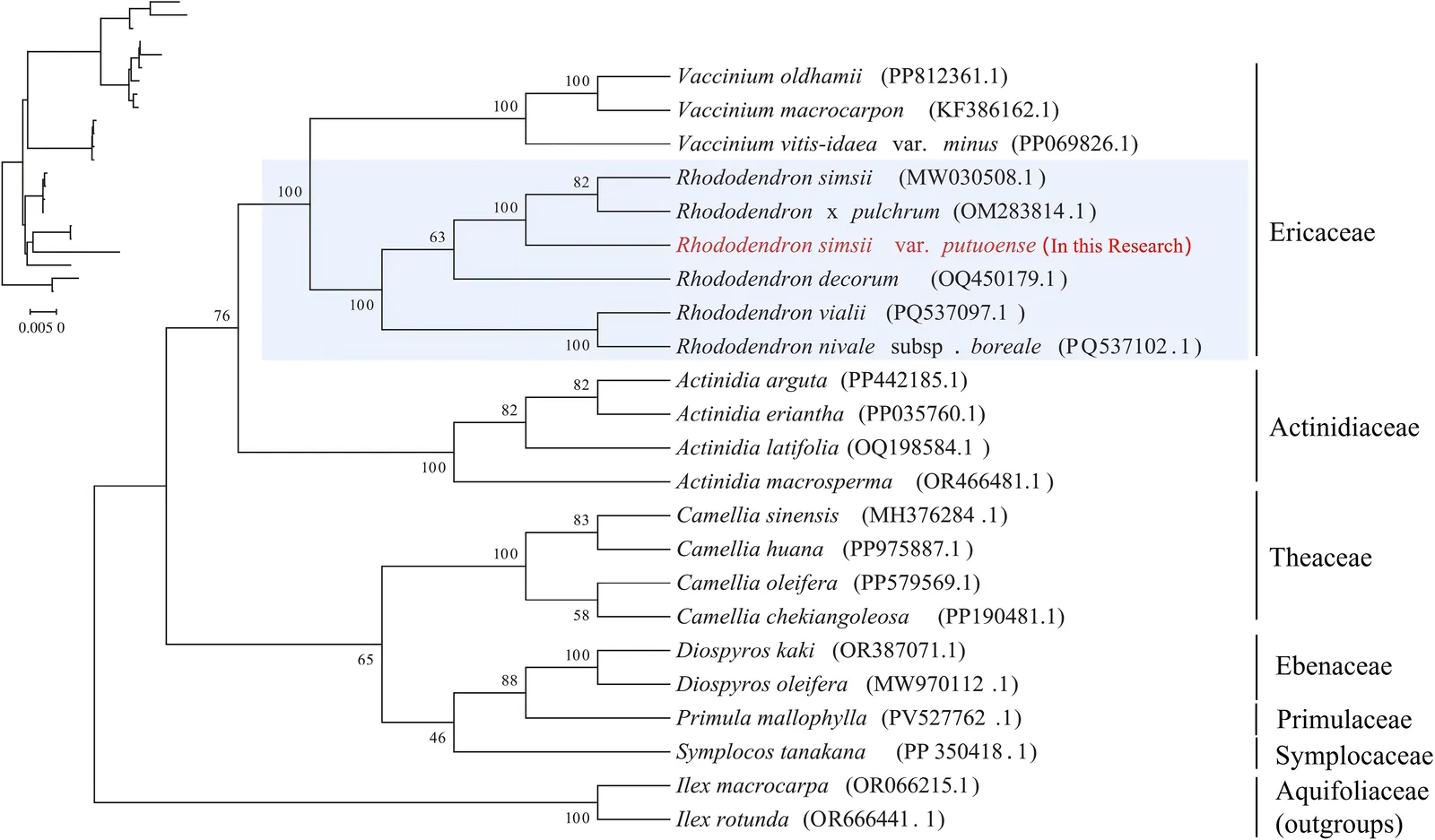
Phylogenetic tree of *R. simsii* var. *putuoense* constructed using mt genomes of 22 species. Bootstrap support values for each node are presented as percentages based on 1,000 replicates.

Synteny analysis revealed significant structural variations in the mt genomes between *R. simsii* var. *putuoense* and other species within the same genus (**Figure 8**). Among them, *R. simsii* var. *putuoense* exhibited the highest level of synteny with *R. simsii* (82.97% and 81.78%, respectively), indicating strong genomic conservation and homology. This was followed by *R.* x *pulchrum* (75.85% and 71.82%, respectively). In contrast, the synteny level between *R. simsii* var. *putuoense* and *R. nivale* subsp. *boreale* was markedly the lowest (47.64% and 62.45%, respectively), reflecting extensive genomic rearrangements and greater evolutionary divergence.

**Figure 8:**
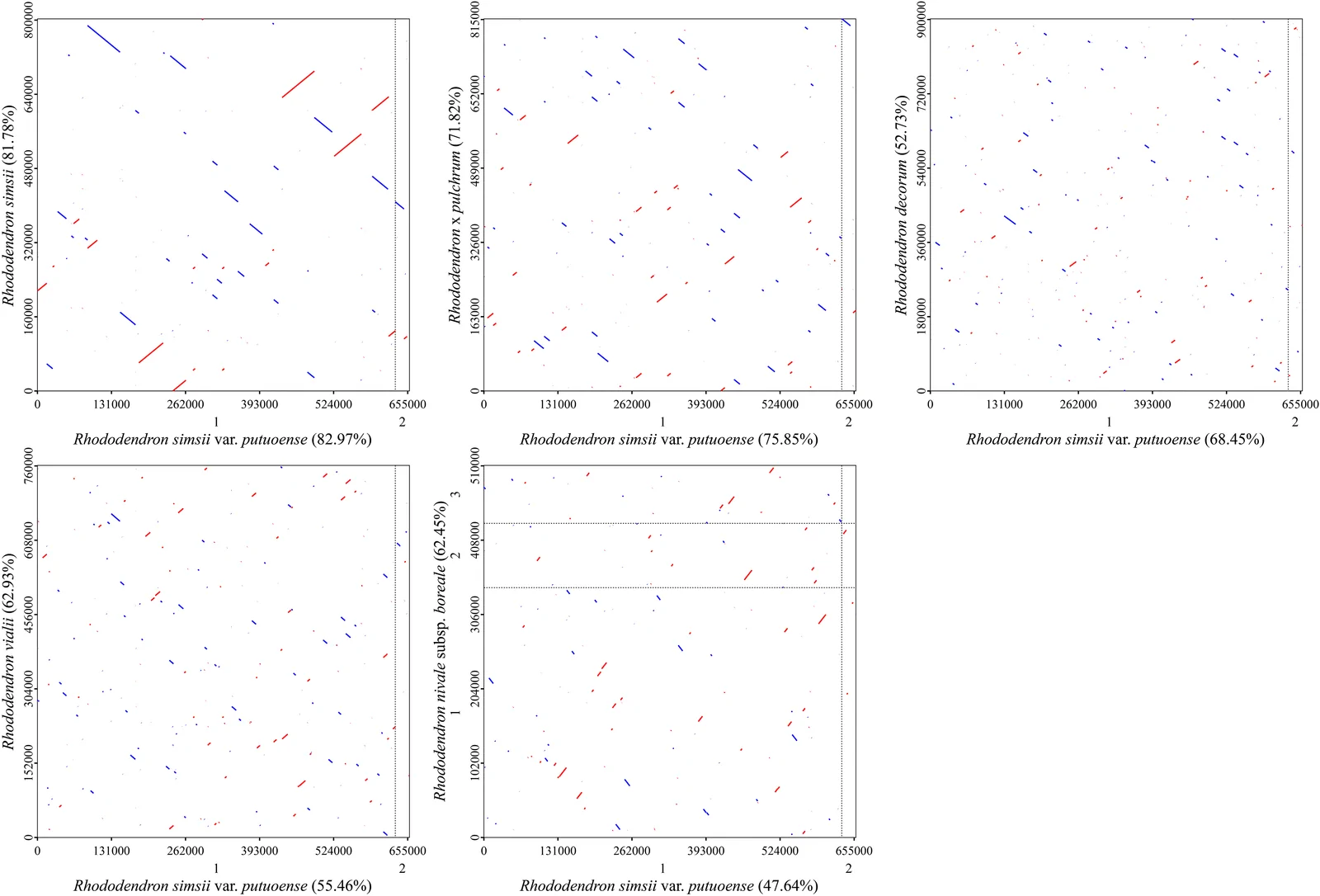
Collinearity analysis of *R. simsii* var. *putuoense* compared to five species in *Rhododendron*. The values in parentheses indicate the proportion of homologous sequences relative to the total genome. The red lines within the box represent forward alignments, and the blue lines represent reverse complementary alignments.

## 4 Discussion

### 4.1 Characteristics of post-transcriptional regulation and evolutionary stability of the mt genome

Mt gene expression in plants is primarily regulated post-transcriptionally, with RNA editing, codon usage bias, and repetitive sequence structures serving as key mechanisms (Binder and Brennicke, 2003; Gualberto and Newton, 2017). In this study, 463 RNA editing sites were predicted in the *R. simsii* var. *putuoense* mtDNA. This number is comparable to those reported for other *Rhododendron* species such as *R. nivale* subsp. *boreale* and *R. vialii* (Lyu et al., 2024), indicating a complete RNA editing system. The observed predominance of hydrophilic-to-hydrophobic transitions after RNA editing is highly significant, as it suggests a concerted mechanism to preserve the hydrophobicity of trans-membrane proteins within the mt respiratory chain (Jobson and Qiu, 2008). Additionally, approximately 0.22% of editing events regulate protein expression by introducing premature stop codons. These precise post-transcriptional regulations compensate for mutations at the genomic level, ensuring the normal execution of mt physiological functions for *R. simsii* var. *putuoense* under complex environments.

Codon usage bias analysis showed that synonymous codons ending in A/U predominate (90.32%) in the *R. simsii* var. *putuoense* mtDNA. This bias is consistent with the typical features of most angiosperm mtDNA, reflecting a balance between mutational pressure and natural selection at the translational level (Smith, 2016). The codon with the highest RSCU value was UUA (1.73), further reinforcing the A/U preference. Leucine, as the most abundant amino acid (11.43%), may be related to its high-frequency distribution in mt transmembrane proteins, such as NADH dehydrogenase subunits.

A total of 527 repetitive sequences were identified in the *R. simsii* var. *putuoense* mtDNA, with dispersed repeats being predominant (72%). The presence of numerous repetitive sequences, particularly forward (F) and palindromic (P) repeats, is generally considered a primary driver of frequent inter-molecular recombination in angiosperm mtDNA (Alverson et al., 2011; Gui et al., 2025). This provides a mechanistic explanation for the multi-branched conformational structure observed in *R. simsii* var. *putuoense* and the substantial synteny variation detected among congeneric species. Similar repeat-mediated structural dynamics have also been reported in *Clausena lansium* ‘Ji Xin’, where dispersed repeats drive mitogenome reorganization into a multipartite architecture (Bi et al., 2026). Notably, such dispersed repeat-mediated recombination primarily operates at the level of large-scale genomic architecture, generating structural polymorphisms among related taxa. In contrast, SSRs and tandem repeats, which together account for the remaining 28% of all identified repeats, are more commonly associated with local sequence variation at finer scales. The relatively low abundance of these small-scale repetitive elements suggests a reduced propensity for localized instability, although this does not preclude the extensive macro-rearrangements driven by the abundant dispersed repeats described above. These two classes of repeats thus contribute to mt genome evolution at distinct structural levels—dispersed repeats shaping global genome architecture through homologous recombination, and SSRs/tandem repeats influencing local sequence dynamics. Furthermore, dispersed repeats may provide recombination sites for IGT, potentially correlating with the six mt-cp homologous segments identified in this study.

In summary, the *R. simsii* var. *putuoense* mt genome exhibits typical characteristics of angiosperm mitochondria in terms of RNA editing, codon usage, and repeat structure. Simultaneously, it displays regulatory features potentially related to its coastal habitat in specific details, such as changes in post-editing amino acid properties and the predominance of dispersed repeats. The combination of RNA editing, codon bias, and repeat-mediated recombination constitutes a multi-layered regulatory diversity that may facilitate phenotypic plasticity in response to coastal environmental fluctuations. These post-transcriptional regulatory mechanisms collectively ensure the accuracy and stability of mt gene expression, providing a vital foundation for the proper assembly and function of respiratory chain complexes.

### 4.2 Evolutionary pressure and habitat adaptation of the mtDNA

In angiosperm mt genomes, *matR* is the only retained intron-encoded maturase gene. Although its reverse transcriptase and endonuclease domains have degenerated during evolution, it maintains a highly conserved X domain (Brown et al., 2014). Despite this structural reduction, *matR* remains widespread across land plants, and its transcripts undergo extensive RNA editing that restores essential amino acid residues, suggesting that it still plays a critical biological role in plant mt functions. For instance, it is required for the splicing of various group II introns (Sultan et al., 2016). Notably, the utility of *matR* as a robust phylogenetic marker is well-established in angiosperms and other seed plants. Previous studies have shown that, compared to plastid genes like *rbcL* and *atpB*, the slowly evolving *matR* gene produces substitutions that are less homoplasious yet highly informative (Zhu et al., 2007). The positive selection on *matR* observed in this study could be interpreted as an instance of adaptive molecular diversity arising from habitat-specific pressures, contributing to the overall genomic diversity of the genus. Our *K*_a_/*K*_s_ analysis revealed that while the majority of PCGs in the *R. simsii* var. *putuoense* mtDNA are under purifying selection to maintain core physiological stability, the *matR* gene exhibited a *K*_a_/*K*_s_ ratio greater than 1. Elevated *K*_a_/*K*_s_ ratios in organellar genes have also been observed in other *Rhododendron* species adapted to harsh habitats (Lyu et al., 2024). However, this gene-wide result should be interpreted cautiously, as a pairwise *K*_a_/*K*_s_ > 1 averages rates across all codons and cannot definitively prove positive selection or account for genetic drift. In contrast, linking this signature directly to coastal adaptations (e.g., salinity or wind stress) remains speculative when based on a single variety. Rather than conclusive proof, the elevated *K*_a_/*K*_s_ in *matR* is a preliminary hint of lineage-specific divergence. Rigorous confirmation requires site-specific codon models, population-level sampling, and functional assays. Overall, these findings provide a valuable baseline for future ecological and functional studies.

### 4.3 Interorganellar horizontal gene transfer and its evolutionary significance

In eukaryotic cells, DNA transfer between mt and cp is a major driving force for plant genome evolution, a process known as IGT (Leister, 2005; Kleine et al., 2009). It represents a specific form of intracellular horizontal gene transfer (HGT) occurring at the organellar level (Filip and Skuza, 2021). Compared to some species with massive plastid-to-mitochondrion DNA transfers (Park et al., 2020), the 0.91% proportion in *R. simsii* var. *putuoense* suggests a relatively stable genomic boundary, possibly reflecting a lineage-specific pattern of organellar co-evolution. Notably, among the six identified homologous segments, five are complete tRNA genes. This high enrichment of tRNA genes is not random but reflects a functional compensation mechanism of the mt genome. Since native mt tRNA genes are frequently lost during evolution, acquiring cp-derived tRNAs through IGT can maintain the integrity of the mt translation system (Gualberto and Newton, 2017). These transferred tRNA segments retain structural integrity in the *R. simsii* var. *putuoense* mtDNA, suggesting they may remain transcriptionally active and participate in mt protein synthesis. In addition to tRNAs, a partially transferred ribosomal RNA gene (*rrn18*) was also identified. Such large-segment transfers typically involve complex genomic recombination events. These homologous sequences not only increase the size of the mt genome but may also serve as sites for non-homologous recombination, further driving structural variations in the mt genome. Combined with the significant structural differences identified in the synteny analysis (**Figure 8**), it can be inferred that these cross-organellar sequence transfers are one of the important drivers leading to the high polymorphism and structural complexity of *Rhododendron* mtDNA.

### 4.4 Phylogenetic relationships and taxonomic status

Given the high species diversity and frequent hybridization within *Rhododendron*, which complicate its taxonomic classification, mtDNA sequences provide a valuable tool for phylogenetic reconstruction. In this study, a ML phylogenetic tree was reconstructed based on complete mt genome sequences. The results showed that *R. simsii* var. *putuoense* clustered with *R. simsii* and *R.* x *pulchrum* into a single clade with 100% bootstrap support. This outcome provides molecular-level support for the taxonomic treatment of *R. simsii* var. *putuoense* as a variety of *R. simsii*. This finding is also congruent with previous mtDNA studies (Shen et al., 2022), which reported that *R. simsii* is the sister species to *R.* × *pulchrum* based on mt genome phylogeny. However, given that the mtDNA is typically uniparentally inherited, this evidence primarily reflects maternal lineage and is insufficient to definitively confirm the parental origin of *R.* x *pulchrum.* Future studies incorporating biparental nuclear markers are necessary to provide a more comprehensive understanding of its hybrid parentage. Synteny analysis (**Figure 8**) further supports this phylogenetic relationship. *R. simsii* var. *putuoense* exhibited the highest synteny proportion with *R. simsii*, followed by *R.* x *pulchrum*, indicating a high degree of genomic structural conservation among the three. In contrast, the synteny proportion between *R. simsii* var. *putuoense* and *R. nivale* subsp. *boreale* was significantly lower, reflecting substantial genomic rearrangement and evolutionary divergence, which is consistent with their positions in different sub-clades on the phylogenetic tree. Taken together, the mitogenomic evidence provides a new perspective on the evolutionary divergence between *R. simsii* var. *putuoense* and its close relative, *R. simsii*. While their high sequence similarity confirms a recent common ancestry, the distinct selective signatures, particularly the adaptive evolution of the *matR* gene discussed in Section 4.2, highlight a clear trajectory of niche differentiation. This molecular divergence aligns with the specialized coastal island habitat of *R. simsii* var. *putuoense*, suggesting that intense environmental pressures have bypassed neutral genetic drift to drive functional adaptations in the mt genome. Consequently, these findings suggest that *R. simsii* var. *putuoense* has developed a unique genetic identity at the organellar level, supporting its status as a distinct evolutionary unit shaped by ecological specialization. Understanding the mitogenomic diversity of *R. simsii* var. *putuoense* not only clarifies its evolutionary distinctiveness but also underscores the importance of preserving genetically unique populations for maintaining regional biodiversity. However, the taxon sampling of *Rhododendron* mt genomes in this study was limited, particularly regarding *R. simsii* varieties and closely related taxa. Future studies should broaden sampling to rigorously resolve the taxonomic status of *R. simsii* var. *putuoense* and clarify its evolutionary relationships within the genus.

## 5 Conclusions

In this study, we report the first complete assembly and annotation of the *R. simsii* var. *putuoense* mt genome, unveiling a multi-branched conformational architecture composed of two linear subgenomic molecules totaling 658,161 bp in length. The genome exhibits typical characteristics of angiosperm mt genomes in gene composition, RNA editing, codon usage bias, and repetitive sequence distribution. Importantly, it also reveals evolutionary dynamics, including IGT and sequence divergence in the *matR* gene, which provide preliminary insights into the mitogenomic variability of this coastal endemic taxon. Phylogenetic and synteny analyses further confirm its close relationship with *R. simsii*, while the distinct selective pressures reveal a trajectory of ecological specialization. In summary, our findings not only illuminate the structural and evolutionary complexity of this mt genome but also highlight the conservation value of lineage-specific genomic diversity, providing a critical genetic foundation for preserving the adaptive potential of this variety and guiding future explorations of cyto-nuclear coevolution within the genus *Rhododendron*.

## Data Availability

The datasets generated during and/or analyzed during the current study are available in the NCBI repository (PZ910021-PZ910022).
